# LiDAR Dynamic Target Detection Based on Multidimensional Features

**DOI:** 10.3390/s24051369

**Published:** 2024-02-20

**Authors:** Aigong Xu, Jiaxin Gao, Xin Sui, Changqiang Wang, Zhengxu Shi

**Affiliations:** School of Geomatics, Liaoning Technical University, Fuxin 123000, China; xuaigong@lntu.edu.cn (A.X.); suixin@lntu.edu.cn (X.S.); 471810032@stu.lntu.edu.cn (C.W.); 471920551@stu.lntu.edu.cn (Z.S.)

**Keywords:** LiDAR dynamic target detection, ICP, RANSAC, XGBoost, Spearman’s rank correlation coefficient, feature screening, sliding window, Boyer–Moore voting

## Abstract

To address the limitations of LiDAR dynamic target detection methods, which often require heuristic thresholding, indirect computational assistance, supplementary sensor data, or postdetection, we propose an innovative method based on multidimensional features. Using the differences between the positions and geometric structures of point cloud clusters scanned by the same target in adjacent frame point clouds, the motion states of the point cloud clusters are comprehensively evaluated. To enable the automatic precision pairing of point cloud clusters from adjacent frames of the same target, a double registration algorithm is proposed for point cloud cluster centroids. The iterative closest point (ICP) algorithm is employed for approximate interframe pose estimation during coarse registration. The random sample consensus (RANSAC) and four-parameter transformation algorithms are employed to obtain precise interframe pose relations during fine registration. These processes standardize the coordinate systems of adjacent point clouds and facilitate the association of point cloud clusters from the same target. Based on the paired point cloud cluster, a classification feature system is used to construct the XGBoost decision tree. To enhance the XGBoost training efficiency, a Spearman’s rank correlation coefficient-bidirectional search for a dimensionality reduction algorithm is proposed to expedite the optimal classification feature subset construction. After preliminary outcomes are generated by XGBoost, a double Boyer–Moore voting-sliding window algorithm is proposed to refine the final LiDAR dynamic target detection accuracy. To validate the efficacy and efficiency of our method in LiDAR dynamic target detection, an experimental platform is established. Real-world data are collected and pertinent experiments are designed. The experimental results illustrate the soundness of our method. The LiDAR dynamic target correct detection rate is 92.41%, the static target error detection rate is 1.43%, and the detection efficiency is 0.0299 s. Our method exhibits notable advantages over open-source comparative methods, achieving highly efficient and precise LiDAR dynamic target detection.

## 1. Introduction

As society enters the era of the mobile Internet, the demand for location services is no longer limited to regional positioning; instead, an all-round positioning service that is not subject to environmental constraints is needed. In outdoor open areas, global navigation satellite systems (GNSSs) can provide mature location services, but these systems have many limitations and deficiencies in complex outdoor environments and indoor environments. Considering these problems, simultaneous localization and mapping (SLAM) has been proposed to promote the gradual maturity of indoor and outdoor integrated high-precision positioning and high-quality spatial data acquisition [1]. Among them, simultaneous localization and mapping based on light detection and ranging (LiDAR SLAM) has the advantages of intuitive mapping, high precision, and the ability to work in all weather conditions [2]. It is widely used in resource exploration, urban planning, agricultural and forestry development, mining inspections, atmospheric detection, and other fields [3,4,5,6]. However, practical application scenes often contain dynamic targets. As one of the key steps of LiDAR SLAM, point cloud interframe registration requires the scanning environment to be completely static. Therefore, the point clouds scanned by dynamic targets have different degrees of influence on LiDAR SLAM. If the dynamic target accounts for a large proportion, the SLAM positioning result will have a large error; if the proportion of dynamic targets is relatively small, the dynamic target will interfere less with the accuracy of the point cloud registration, but the point cloud scanned during its movement will leave traces in the final point cloud map, decreasing the mapping quality. Dynamic point clouds not only affect the effectiveness of the above positioning and navigation methods, but also cannot be ignored in the geographic information system (GIS) and remote sensing (RS) fields. If the LiDAR point cloud used to construct a geospatial database or generate a digital elevation model (DEM) contains many dynamic point clouds, the point cloud splicing accuracy and the subsequent application of the database and model will be impacted. When LiDAR point clouds are used for agricultural and forestry census and geological monitoring, there will be a certain degree of error in the corresponding results, because dynamic targets rarely have periodic reproducibility. When LiDAR point clouds are used for building modelling, urban planning, and other tasks, the use of point clouds scanned by dynamic targets does not have research significance; therefore, removing these clouds in advance can effectively improve the efficiency of data processing. In summary, efficiently and accurately detecting and eliminating LiDAR dynamic point clouds are issues of widespread concern in various fields; these tasks are the focus of this paper.

To address the problem of LiDAR dynamic target detection, commonly used methods can be divided into three main categories: segmentation-based methods, visibility-based methods, and voxel-based methods. Segmentation-based methods can be further divided into methods based on traditional clustering segmentation and methods based on learning segmentation. Methods based on traditional clustering segmentation generally use region growing [7], fast point feature histograms (FPFHs) [8], and other algorithms for point cloud clustering segmentation, and then, through point cloud direct registration [9], with the help of other sensors [10], multisource information [11,12], and other methods to unify the adjacent frame point cloud coordinate system, compare the indicators used to judge the motion state of the point cloud cluster and the corresponding threshold to detect dynamic targets. These methods ensure a low data processing complexity, but typically use a single index as the benchmark to judge the motion state of the target, and different thresholds must be set for the corresponding indices in different scenes to obtain effective detection results. Methods based on learning segmentation generally use 3D−MiniNet, RangeNet++, and other networks to directly semantically segment LiDAR point clouds [13,14] or project point clouds into two-dimensional images for indirect semantic segmentation [15,16,17] and use the obtained semantic labels to detect dynamic targets. These methods are convenient to use and have a wide range of applications. However, constructing a suitable training set, reducing the workload of the training set construction, and improving the training efficiency and accuracy are still key challenges when using these methods. Visibility-based methods are generally based on the physical premise that ‘light propagates along a straight line’. The 3D LiDAR point cloud is projected into a 2D distance image [18] or depth image [19]. The single-frame point cloud image is aligned to the corresponding position in the overall point cloud image through perspective coordinate transformation, and the residual image is generated by the pixel difference for dynamic target detection [20]. This kind of method does not require pretraining and is not restricted by the category or number of dynamic targets. However, processing each frame point cloud requires a perspective coordinate transformation, so this kind of method relies on high-precision pose transformation parameters. This method is applicable only to sparse LiDAR point clouds; otherwise, the data processing efficiency is extremely low. In addition, at present, this kind of method still faces two key problems: laser beam physical characteristic interference and static point invisibility. The former is caused by the interference of the point cloud projection effect caused by special LiDAR laser beam structures, such as parallelism and occlusion. The latter problem is caused by the presence of dynamic targets that maintain the same frequency movement as LiDAR during data acquisition. The same frequency target continuously blocks the point cloud behind it; as a result, detecting it effectively through the residual image is impossible. Voxel-based methods can be further divided into probability statistics methods and descriptor comparison methods. Probability statistics methods [21] are also based on the physical premise that ‘light propagates along a straight line’. The 3D environment is projected onto the 2D plane and divided into several small grids. As the LiDAR point cloud to be detected accumulates frame by frame, the ray casting-based (RC) algorithm is used to identify the situation where each grid is hit and crossed, and the probability that the grid contains dynamic targets is calculated to screen the dynamic grid. This kind of method achieves the batch detection of the point cloud motion state with the grid as the smallest unit. However, when given a large incident angle of a laser beam or occlusion, the detection results easily become abnormal. Furthermore, detecting each frame point cloud requires traversing all the grids passed by each laser beam, which consumes a large amount of computing resources. The descriptor comparison method requires constructing a global point cloud map or a local point cloud map in advance during the data acquisition process to form a prior reference basis [22,23]. The prior point cloud map and the point cloud to be detected are rasterized. Whether the descriptors of the paired grids differ is determined, and the nonground points in significantly different grids are regarded as dynamic point clouds. This kind of method can effectively detect dynamic targets in various states, but requires a prior map as a reference benchmark; as a result, it is generally used for postdetection, which limits its application. In addition, the grid size directly impacts the detection effect of voxel-based methods. If the grid is too large, part of the static point cloud will be removed, and if the grid is too small, the data processing efficiency will be significantly reduced. In summary, each method has advantages and limitations. It is necessary to study in depth a LiDAR dynamic target detection method with universal applicability that accounts for both detection accuracy and data processing efficiency.

To reduce the influence of heuristic thresholds and auxiliary processes such as point cloud projection and grid segmentation on the final LiDAR dynamic target detection results, based on the point cloud clustering results and the unified adjacent frame point cloud coordinate system, the multidimensional position and geometric structure differences between the paired point cloud clusters are comprehensively considered in this paper. By extracting the characteristics of the point cloud cluster and using a machine learning method to detect the motion state of each point cloud cluster in each frame point cloud, high-precision and high-efficiency LiDAR dynamic target detection is achieved. Among many machine learning algorithms, XGBoost [24] has the advantages of flexibility, accuracy, and efficiency and has been optimized by relevant experts and scholars from the perspectives of data processing, multilabel classification, and hyperparameter tuning [25,26,27,28]. Therefore, this algorithm is widely used to address various classification and regression problems [29,30,31]. However, it is rarely used to detect LiDAR dynamic targets. Therefore, it is taken as the core algorithm of LiDAR target motion state detection in this paper. Compared with other learning-based LiDAR dynamic target detection methods, our method does not require a complex training network structure, training the XGBoost decision tree by reasonably constructing an optimal classification feature subset to detect LiDAR dynamic targets. The proposed method has universal applicability to different environments. When the optimal classification feature subset is constructed, effective feature screening and dimensionality reduction are conducted, which ensures the classification accuracy, compresses the optimal feature subset dimension, and considers the model training efficiency. In addition, the classification label acquisition and classification feature quantification of the training dataset adopt a fully automatic mode, which avoids introducing human error.

The main contributions of this paper are as follows.

- Based on the clustering results of LiDAR point cloud clusters, the geometric center point set of the point cloud clusters of each frame point cloud is taken as the research object, and a double registration algorithm suitable for sparse point clouds is proposed. In the coarse registration stage, the iterative closest point (ICP) algorithm is used to obtain the rough pose relationship between the geometric center point sets of adjacent frame point cloud clusters. In the fine registration stage, the random sample consensus (RANSAC) algorithm and the four-parameter coordinate transformation algorithm are used to calculate a more accurate pose relationship.
- The above pose relationship is used to unify the coordinate datum between the geometric center point sets of adjacent frame point cloud clusters; then, the matching results of the point cloud clusters scanned by the same target are obtained, and the multidimensional position and geometric structure differences are calculated to construct the point cloud cluster classification feature system. Since point cloud cluster features are generally quantitative features, the number of feature splittings (weight) is taken as an indicator of the importance of the features. Moreover, based on Spearman’s rank correlation coefficient (SCC), the optimal classification feature subset is constructed by bidirectional feature screening and dimensionality reduction to facilitate both accurate detection and training efficiency for XGBoost.
- Considering that machine learning algorithms have certain mechanical properties and cannot detect dynamic targets with special states, a double Boyer–Moore voting-sliding window scheme based on the sliding window (SW) strategy and the Boyer–Moore voting (BMV) strategy is designed to achieve the secondary correction of the preliminary detection results of XGBoost, thereby improving the accuracy of the final LiDAR dynamic target detection.
- In the corresponding experiments, the effectiveness of the proposed main algorithms is reasonably verified, and it is successfully verified that our method can effectively detect the motion state based on the multidimensional features of adjacent frame paired point cloud clusters, so as to achieve accurate and efficient LiDAR dynamic target detection.

The remainder of this paper is organized as follows. In Section 2.1, the framework of the entire method is outlined. In Section 2.2, a double registration algorithm is proposed to ensure that the point cloud clusters scanned by the same target in two adjacent frames are accurately matched. The extraction and quantification processes of the corresponding classification feature system and the training set classification label when using XGBoost for LiDAR dynamic target detection are described in Section 2.3. In Section 2.4, XGBoost is improved from the perspectives of both model detection accuracy and training efficiency, and a double Boyer–Moore voting-sliding window is designed to correct the preliminary detection results. The detailed experimental setup and discussion are reported and analyzed in Section 3. Finally, in Section 4, the work of this paper is summarized, and the advantages and disadvantages of our method are discussed and delineated.

## 2. Methodology

In this paper, we use the superscript T to denote the transpose of a vector or matrix, with lowercase bold symbols (e.g., η) denoting vectors and uppercase bold symbols (e.g., T) denoting matrices and collections. For any vector η, $\left\|\eta \right\|$ denotes its Euclidean norm.

### 2.1. Method Overview

**Figure 1:**
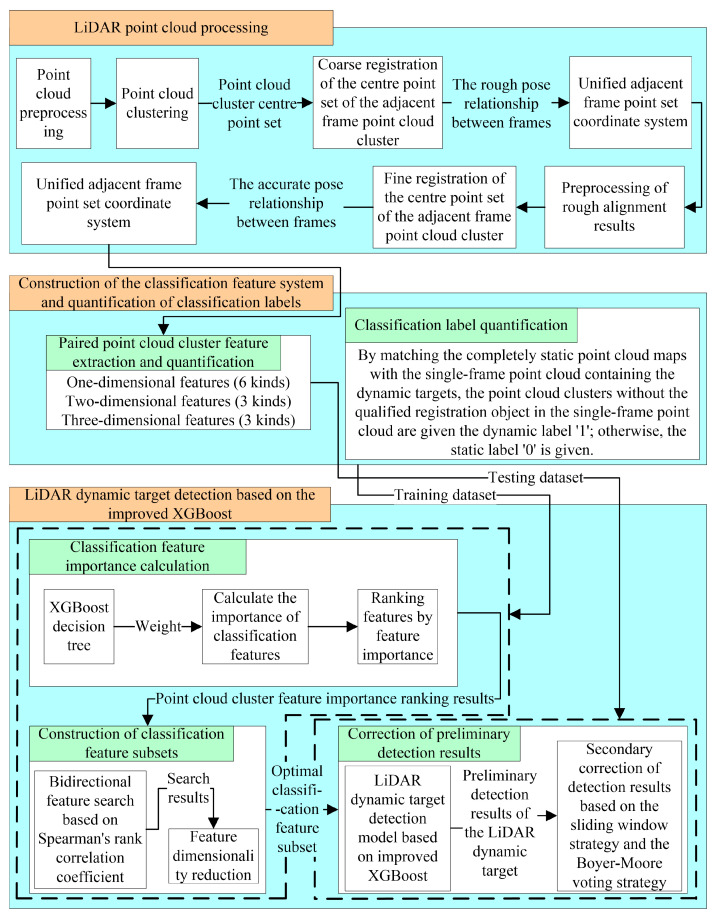
Flowchart of the LiDAR dynamic target detection method based on multidimensional features.

The proposed LiDAR dynamic target detection method based on multidimensional features is shown in Figure 1. The main framework consists of three parts: LiDAR point cloud processing, the construction of a classification feature system and quantification of classification labels, and LiDAR dynamic target detection based on improved XGBoost.

The LiDAR point cloud processing procedure is shown in the first part of Figure 1. Point cloud preprocessing and point cloud cluster segmentation are conducted, and a double registration algorithm suitable for sparse point clouds is proposed to obtain accurate adjacent frame point cloud cluster matching results.

The construction of the classification feature system and the quantification of the classification labels are shown in the second part of Figure 1. The extraction and quantification of differences between the multidimensional positions and geometric structures of the paired point cloud clusters are determined, and a quantification method for classification labels is used to construct the model training dataset.

The LiDAR dynamic target detection process based on improved XGBoost is shown in the third part of Figure 1. In this process, based on traditional XGBoost, weight is used as an indicator of the importance of the features, and a strategy for efficiently constructing an optimal classification feature subset is proposed that considers the training efficiency and detection accuracy of the model. In addition, a secondary correction strategy for the preliminary detection results of XGBoost is proposed to improve the final LiDAR dynamic target detection accuracy.

### 2.2. LiDAR Point Cloud Processing

#### 2.2.1. Construction of the Point Cloud Cluster Center Point Set

LiDAR point clouds exhibit wide coverage, a high measurement accuracy, and dense data, but they also exhibit problems such as equipment system errors [32], point cloud motion distortions [33], and including noise points and discrete points [34]. Therefore, data preprocessing must be performed before LiDAR point clouds can be used for related work. In this paper, an unsupervised LiDAR point cloud optimization algorithm is used to estimate and compensate for system errors in the equipment, and the velocity updating-iterative closest point (V−ICP) method is used to remove point cloud motion distortion [35]. These processing steps greatly improve the quality of the LiDAR point clouds. However, since the purpose of this study is to efficiently detect LiDAR dynamic targets, the high density of the point clouds leads to heavy data processing tasks, so point cloud downsampling is needed. Generally, a point cloud is downsampled by point cloud filtering, removing the noise points and discrete points [36]. Commonly used downsampling methods include Gaussian filtering, direct filtering, voxel filtering, statistical filtering, and bilateral filtering. The effect of direct filtering depends on the heuristic threshold [37]. Gaussian filtering is applicable only to point clouds that follow a normal distribution [38], bilateral filtering is applicable only to ordered point clouds [39], and statistical filtering requires a large amount of calculation [40]. As a result, the voxel filtering method is used in this paper. Constructing a voxel group composed of several three-dimensional hexahedrons ensures that all the LiDAR point clouds of the corresponding frame are completely covered. The number of laser points in each small voxel is counted. The voxels with numbers less than the set threshold are regarded as sparse voxels, and their laser points are regarded as discrete points or noise points and eliminated. For the remaining voxels, the mean value of the point cloud coordinates is calculated separately and used as the voxel center of gravity to replace all the laser points in the current voxel, achieving point cloud downsampling.

Based on the preprocessed LiDAR point cloud, ground segmentation, point cloud cluster clustering, and point cloud cluster geometric center coordinate calculations are conducted using the method provided in Reference [41] to construct the point cloud cluster center point set corresponding to each frame of the LiDAR point cloud. The point cloud cluster center point set corresponding to the frame point cloud is recorded as $E_{i}$.

#### 2.2.2. Coarse Registration of the Center Point Set of the Adjacent Frame Point Cloud Cluster

The purpose of this study is to judge the motion state of a point cloud cluster based on the multidimensional position and geometric structure difference between the point cloud clusters generated by the same target scanned in the adjacent frame LiDAR point cloud. Therefore, the point cloud cluster matching results generated by the same target scanned from the adjacent frame point cloud cluster set must be accurately obtained. In this paper, this task is equivalent to registering the center point set of the adjacent frame point cloud cluster with high precision, and the point cloud cluster pairing result is obtained based on the registration result of the center point set.

**Figure 2:**
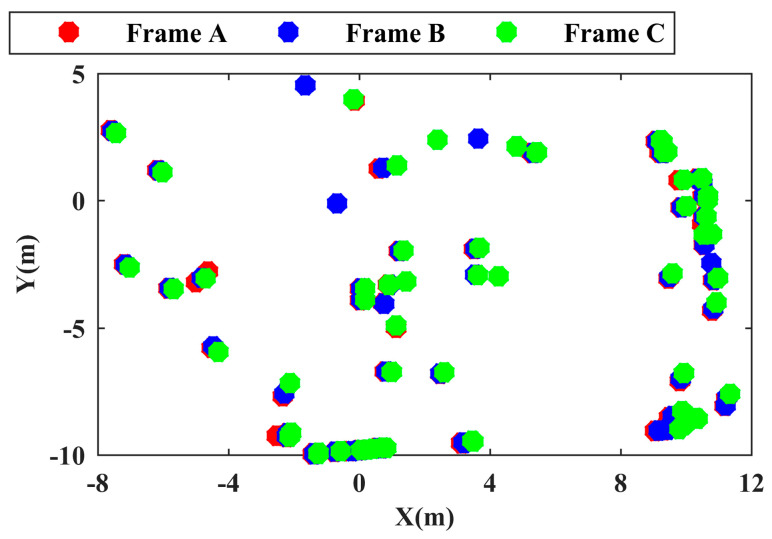
Distribution of point cloud cluster centroid sets for different frames.

There are often differences between the degrees of position and pose differences of different frame point cloud cluster center point sets that do not pass through the unified coordinate system. As shown in Figure 2, three color point cloud cluster center point sets correspond to three different LiDAR point clouds. Frame A and frame B are adjacent frames, and five frame intervals exist between frame C and frame A. As shown, the more the frames are separated, the more the overall pose differs between the point cloud cluster center point sets. Taking frame A and frame B as examples, if no registration processing is performed and the matching objects of each point in $E_{B}$ are searched directly in $E_{A}$, the probability of incorrect matching results is great. In addition, due to the lack of a unified coordinate system, the difference in the position of the point cloud cluster calculated at this time does not have any research significance and cannot be used to judge the motion state of the point cloud cluster. Therefore, a double registration method for the center point set of the point cloud clusters is proposed to accurately solve the pose relationship between $E_{A}$ and $E_{B}$. The double registration method for the point cloud consists of two parts: coarse registration and fine registration. In this section, the coarse registration process is proposed.

**Figure 3:**
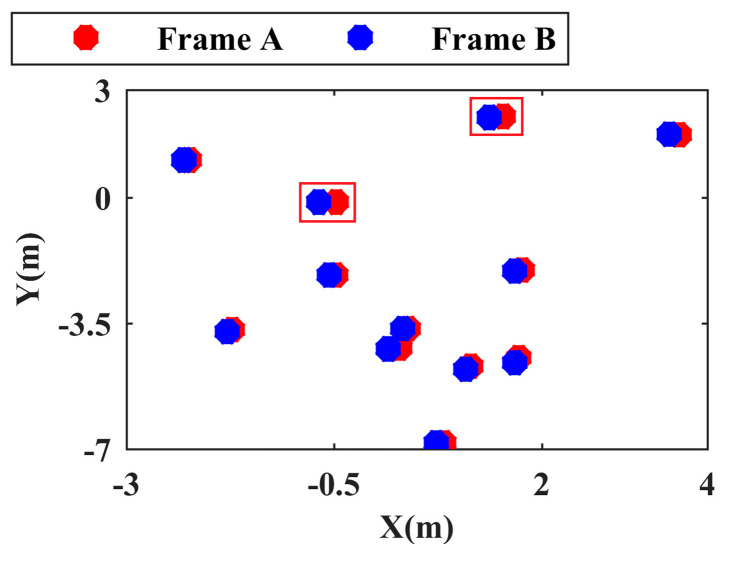
Distribution of the cluster centroids of two adjacent frames in a unified coordinate system after coarse registration.

Because there is no dramatic change in the pose between frame A and frame B and because the number of points in the center point set of the point cloud cluster is much smaller than the number of points in the entire point cloud, the initial pose matrix of the ICP algorithm [42] is set to a unit matrix, and $E_{A}$ and $E_{B}$ are coarsely registered quickly. Based on the rough pose relationship between frames obtained by coarse registration, $E_{B}$ is converted to the $E_{A}$ coordinate system. By setting the appropriate search radius, each point in $E_{B}$ is taken as the research object, and a nearest neighbor search is performed in $E_{A}$. The point cloud cluster corresponding to the point without search results is recorded as the ’missing’ point cloud cluster. Similarly, using the same search radius, each point in $E_{A}$ is taken as the research object, and the ’missing’ point cloud cluster is reverse-searched in $E_{B}$. In addition, after coarse registration and application of the unified coordinate system, the nearest neighbor matching object of each point in the current $E_{B}$ can be obtained as shown in $E_{A}$. To facilitate an intuitive display, in Figure 3, the matching results of some points are shown, and the object shown in the red box is the center point of the known dynamic point cloud cluster.

Because ICP iteratively registers the nearest point and calculates the overall registration error to obtain the optimal solution, it cannot intelligently distinguish the dynamic point cloud cluster center point and the static point cloud cluster center point in the set E; as a result, the registration error of the dynamic point cloud cluster center point impacts the overall registration result. As shown in Figure 3, these problems lead to different degrees of positional differences between the center points of the paired static point cloud clusters after coarse registration. Therefore, the position difference calculated based on the center point of the nearest paired point cloud cluster after coarse registration cannot be used to judge the motion state of the point cloud cluster, and the calculation accuracy of the interframe pose relationship must be further improved.

#### 2.2.3. Fine Registration of the Center Point Set of the Adjacent Frame Point Cloud Cluster

Because the LiDAR laser beam propagates along a straight line, the geometric structure difference largely differs between the point cloud clusters generated by the same target in frame A and frame B due to occlusion or scanning angle changes, which leads to a large degree of positional difference between the center points of the corresponding paired point cloud clusters of some static targets after rough registration. This kind of point cloud cluster is recorded as a pseudodynamic point cloud cluster. To prevent these pseudodynamic point cloud clusters from affecting the fine registration process, the coarse registration results of $E_{A}$ and $E_{B}$ are preprocessed, and the center point of the pseudodynamic point cloud cluster is filtered in advance to prevent it from being used as the input for fine registration.

Based on the geometric structure characteristics of each point cloud cluster, the description vector η is constructed as follows:

(1) $$\eta ={\left[\begin{array}{cccc} \varphi_{1} & \varphi_{2} & \varphi_{3} & \varphi_{4} \end{array}\right]}^{T}$$

where $\varphi_{1}$ represents the X-axis span of the point cloud cluster, $\varphi_{2}$ represents the Y-axis span of the point cloud cluster, $\varphi_{3}$ represents the Z-axis span of the point cloud cluster, and $\varphi_{4}$ represents the number of laser points in the point cloud cluster.

The nearest neighbor pairing results of the point cloud clusters after coarse registration are transformed into descriptive vectors in pairs, and the cosine similarity S [43] between vectors is calculated as follows:

(2) $$S=\left(\eta_{A}^{T}\cdot \eta_{B}^{k}\right)/\left(\left\|\eta_{A}\right\|\left\|\eta_{B}^{k}\right\|\right)=\left(\sum_{j=1}^{4}\left(\varphi_{A}^{j}\varphi_{B}^{kj}\right)\right)/\left(\sqrt{\sum_{j=1}^{4}{\left(\varphi_{A}^{j}\right)}^{2}}\sqrt{\sum_{j=1}^{4}{\left(\varphi_{B}^{kj}\right)}^{2}}\right)$$

where $\eta_{B}^{k}$ represents the description vector of the k-th point cloud cluster in $E_{B}$, $\eta_{A}$ represents the description vector of the paired point cloud cluster in $E_{A}$, and j represents the number of elements in the description vector. If S is less than the set threshold, the point cloud cluster being processed in $E_{B}$ is recorded as pseudodynamic. The above steps are repeated to effectively screen all pseudodynamic point cloud clusters in $E_{B}$. If there is no geometric structure change between the matching results of the adjacent frame dynamic point cloud clusters, the similarity between the corresponding description vectors must be greater than the set threshold; therefore, the above process cannot effectively filter the dynamic point cloud clusters.

**Figure 4:**
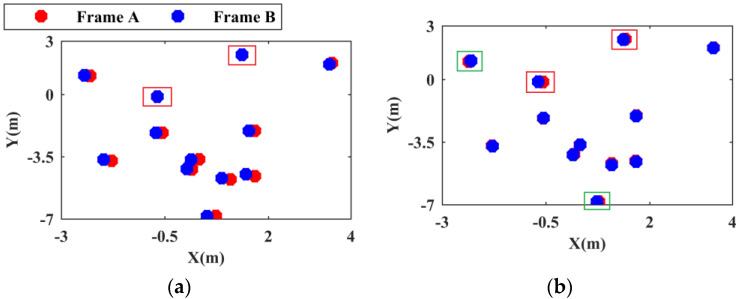
Distribution of the cluster centroids of two adjacent frames in a uniform coordinate system after fine registration: (**a**) dynamic centroids constituting initial interior points; and (**b**) static centroids constituting initial interior points.

After eliminating the ‘missing’ point cloud clusters and the pseudodynamic point cloud clusters, the center point set of the point cloud cluster is effectively streamlined. Since the pose difference between the point cloud cluster center point sets of frame A and frame B is small after coarse registration and a unified coordinate system is applied, and since several homonymous point pairs are formed by the nearest neighbor pairing of the point cloud cluster center point, four-parameter or seven-parameter coordinate transformation methods can be used for fine registration [44]. In the real scene, there are few targets moving only along the Z-axis direction. The seven-parameter coordinate transformation method requires more calculation than the four-parameter coordinate transformation method. Therefore, in this paper, both $E_{A}$ and $E_{B}$ are projected onto the X−Y plane and the four-parameter coordinate transformation method is used to identify the pose relationship. If all the above homonymous point pairs are used to construct the least squares equation to solve the transformation relationship, the influence of dynamic point cloud clusters on the calculation accuracy is also ignored. Therefore, based on the idea of RANSAC [45], two homonymous point pairs are randomly selected to construct the equation and calculate the transformation relationship T. The selected point cloud cluster center points are called the initial interior points. T is used to perform coordinate transformation on the remaining points, and the coordinate offset between each point in $E_{B}$ after transformation and its matching points is calculated. Points below the set threshold are recorded as interior points, and the number of interior points is counted. When the initial interior points are the center points of the point cloud cluster corresponding to the dynamic target, the conversion relationship is calculated, and coordinate transformation is performed. As shown in the red box in Figure 4a, no offset exists between the initial interior points and their matching points; however, a large offset exists between the remaining points and their matching points, and the number of interior points in this case is very small. As shown in Figure 4b, when the initial interior points are the center points of the point cloud cluster corresponding to the static target, the number of interior points is large. A significant offset exists between the center points of the dynamic point cloud cluster and the matching points shown in the red box in the figure, which is normal; a small offset exists between some static point cloud cluster center points and the matching points shown in the green box, which is caused by the occlusion and parallax described above. The process of selecting the initial interior points and counting the number of interior points is iterated until all the combinations of homonymous point pairs are processed, and the process is then stopped. The corresponding T when the number of interior points is the largest is taken as the fine registration result.

### 2.3. Construction of the Classification Feature System and Quantification of Classification Labels

#### 2.3.1. Paired Point Cloud Cluster Feature Extraction and Quantification

Based on the clustering results of the adjacent frame LiDAR point clouds and the matching results of the adjacent frame point cloud clusters after the double registration of the unified coordinate system, 12 kinds of point cloud cluster features that can comprehensively describe the multidimensional position and geometric structure differences between the paired point cloud clusters are extracted and quantified. These features are used to construct the classification feature system. Following the different geometric dimensions of feature extraction, these features are categorized into one-dimensional features, two-dimensional features, and three-dimensional features.

1. A one-dimensional feature refers to a feature extracted based on the difference in position or geometric structure between the paired point cloud clusters in a single dimension of X, Y, and Z. In this paper, six one-dimensional features are extracted, namely, the X-axis displacement $D_{X}$, Y-axis displacement $D_{Y}$, Z-axis displacement $D_{Z}$, X-axis span change degree $L_{X}$, Y-axis span change degree $L_{Y}$, and Z-axis span change degree $L_{Z}$. The methods used to quantify the above six features are as follows.

Taking the point clouds of frame A and frame B after registration as examples, the center point of the k-th point cloud cluster in $E_{A}$ is denoted as $\left(X_{A}^{k},Y_{A}^{k},Z_{A}^{k}\right)$ and the center point of the t-th point cloud cluster paired with the above point cloud cluster in $E_{B}$ is denoted as $\left(X_{B}^{t},Y_{B}^{t},Z_{B}^{t}\right)$. Using the above variables, $D_{X}$, $D_{Y}$, and $D_{Z}$ are calculated as follows:

(3) $$\begin{array}{ccc} D_{X}=\left|X_{A}^{k}-X_{B}^{t}\right| & D_{Y}=\left|Y_{A}^{k}-Y_{B}^{t}\right| & D_{Z}=\left|Z_{A}^{k}-Z_{B}^{t}\right| \end{array}$$

The maximum and minimum values of the X, Y, and Z three-axis coordinates of each laser point in each point cloud cluster are recorded as $X_{max}$, $X_{min}$, $Y_{max}$, $Y_{min}$, $Z_{max}$, and $Z_{min}$, and the three-axis spans $H_{X}$, $H_{Y}$, and $H_{Z}$ of the point cloud cluster are calculated as follows:

(4) $$\begin{array}{ccc} H_{X}=X_{max}-X_{min} & H_{Y}=Y_{max}-Y_{min} & H_{Z}=Z_{max}-Z_{min} \end{array}$$

Based on the three-axis span of the point cloud cluster, $L_{X}$, $L_{Y}$, and $L_{Z}$ are calculated as follows:

(5) $$\begin{array}{ccc} L_{X}=\left(\left|H_{XA}^{\;\;\;k}-H_{XB}^{\;\;\;t}\right|\right)/H_{XA}^{\;\;\;k} & L_{Y}=\left(\left|H_{YA}^{\;\;\;k}-H_{YB}^{\;\;\;t}\right|\right)/H_{YA}^{\;\;\;k} & L_{Z}=\left(\left|H_{ZA}^{\;\;\;k}-H_{ZB}^{\;\;\;t}\right|\right)/H_{ZA}^{\;\;\;k} \end{array}$$

2. Two-dimensional features are features extracted based on the geometric structure differences between paired point cloud clusters in any two dimensions of X, Y, and Z. In this paper, three two-dimensional features are extracted, namely, the X−Y projection area change degree $S_{XY}$, the X−Z projection area change degree $S_{XZ}$, and the Y−Z projection area change degree $S_{YZ}$. The methods used to quantify the above three features are as follows.

Based on the quantitative results of $H_{X}$, $H_{Y}$, and $H_{Z}$, the projection areas $C_{XY}$, $C_{XZ}$, and $C_{YZ}$ of the point cloud clusters in the X−Y plane, X−Z plane, and Y−Z plane are calculated as follows:

(6) $$\begin{array}{ccc} C_{XY}=H_{X}H_{Y} & C_{XZ}=H_{X}H_{Z} & C_{YZ}=H_{Y}H_{Z} \end{array}$$

Based on the plane projection area of the point cloud cluster, $S_{XY}$, $S_{XZ}$, and $S_{YZ}$ are calculated as follows:

(7) $$\left\{\begin{array}{l} S_{XY}=\left(\left|C_{XYA}^{\;\;\;\;\;\;k}-C_{XYB}^{\;\;\;\;\;\;t}\right|\right)/C_{XYA}^{\;\;\;\;\;\;k}\\ S_{XZ}=\left(\left|C_{XZA}^{\;\;\;\;\;\;k}-C_{XZB}^{\;\;\;\;\;\;t}\right|\right)/C_{XZA}^{\;\;\;\;\;\;k}\\ S_{YZ}=\left(\left|C_{YZA}^{\;\;\;\;\;\;k}-C_{YZB}^{\;\;\;\;\;\;t}\right|\right)/C_{YZA}^{\;\;\;\;\;\;k} \end{array}\right.$$

3. A three-dimensional feature is a feature extracted based on the geometric structure differences between the paired point cloud clusters in the X, Y, and Z dimensions. In this paper, three three-dimensional features are extracted, namely, the degree of change in the volume of the point cloud cluster G, the degree of change in the number of laser points M, and the degree of change in the density of the laser points P. The methods used to quantify the above three features are as follows.

Based on the quantitative results of $H_{X}$, $H_{Y}$, and $H_{Z}$, the volume V of the smallest outsourced parallel regular hexahedron of the point cloud cluster is calculated as follows:

(8) $$V=H_{X}H_{Y}H_{Z}$$

Based on the point cloud cluster outsourcing hexahedral volume, G is calculated as follows:

(9) $$G=\left(\left|V_{A}^{k}-V_{B}^{t}\right|\right)/V_{A}^{k}$$

In the above data processing process, the number of laser points N in each point cloud cluster is directly counted, and M is calculated as follows:

(10) $$M=\left(\left|N_{A}^{k}-N_{B}^{t}\right|\right)/N_{A}^{k}$$

Based on the quantitative results of V and the statistical results of N, the laser point density ρ of the point cloud cluster is calculated as follows:

(11) ρ=N/V

Based on the quantitative results of ρ, P is calculated as follows:

(12) $$P=\left(\left|\rho_{A}^{k}-\rho_{B}^{t}\right|\right)/\rho_{A}^{k}$$

By analyzing the data and calculation methods required to quantify these 12 features, the corresponding point cloud cluster classification feature system can be constructed hierarchically for each frame of the LiDAR point cloud by traversing the point cloud cluster set once and using multithreading processing, which ensures the timeliness of data processing.

#### 2.3.2. Classification Label Quantification

To ensure the universal applicability of the proposed LiDAR dynamic target detection model, LiDAR point clouds are collected in real indoor and outdoor scenes. By constructing an a priori point cloud map and using the descriptor comparison method based on the voxel-based method, the motion state classification label is automatically assigned to each point cloud cluster in a single-frame point cloud. The specific process is as follows.

Two rounds of data acquisition are conducted in each real scene. The first round is conducted in the period with no dynamic targets or very few dynamic targets in each scene and is used to construct an a priori point cloud map as a reference base. The second round is conducted during the period with more dynamic targets in each scene and is used to quantify the classification feature system and classification labels.

For each data acquisition scene, based on the first data collected, the LiDAR Odometry and Mapping (LOAM) algorithm [46] is used to construct an a priori point cloud map. Because the secondary collected LiDAR point cloud encompasses many dynamic targets, errors will arise when directly detecting dynamic targets directly using the scan-to-map approach [47] to register a single-frame point cloud and a point cloud map. To obtain a more accurate training set classification label, the descriptor comparison method is used to detect the motion state of each point cloud cluster in each frame of the LiDAR point cloud by dividing the grid and comparing the descriptors based on the registered single-frame point cloud and the prior map. The dynamic point cloud cluster and the ’missing’ point cloud cluster are given a dynamic label of ‘1’, and the remaining point cloud clusters are given a static label of ‘0’.

### 2.4. LiDAR Dynamic Target Detection Based on the Improved XGBoost

#### 2.4.1. Construction of Classification Feature Subsets

The model training dataset in this paper comprises the above point cloud cluster classification feature system and classification labels. By constructing the objective function and second-order Taylor expansion, the second-order derivative information is used to train the XGBoost classification decision tree, and the model complexity is optimized as a regularization term to ensure a higher model generalizability [48].

The above process outputs three independent feature importance metrics: weight, gain, and cover. Each index can be used as a benchmark for constructing classification feature subsets during model training, which is used to screen important features to improve the classification effect of XGBoost [49]. Because including enumeration features in the point cloud cluster classification feature system constructed in this paper is difficult, weight is selected as the only feature importance measure index, and the features are arranged from large to small according to the weight index, denoted as sequence $F_{ps}$; the arrangement from small to large is denoted as sequence $F_{io}$.

Because the classification feature system contains more features and the number of samples in the training set based on the independent point cloud cluster is large, under the premise of ensuring the detection accuracy of the model, an optimal feature subset construction strategy based on Spearman’s rank correlation coefficient-bidirectional search for dimensionality reduction (SCC−BSDR) is proposed in this paper [50]. This approach is used to improve the training efficiency of the model. There are three traditional feature search strategies [51], namely, forward search, backward search, and bidirectional search. Forward and backward searches easily fall into local optimal solutions. Although the bidirectional search is more reliable, its computational complexity is significantly greater than that of the other search strategies.

In this paper, the optimal feature subset construction is based on the bidirectional feature search strategy. On this basis, Spearman’s rank correlation coefficient is used to improve the construction efficiency and a dimensionality reduction strategy is used to simplify the composition of the feature subset. First, the most important feature is obtained from $F_{ps}$ and added to the feature subset to construct a classification decision tree. The current classification accuracy is calculated and used as the initial classification accuracy benchmark. The Spearman’s rank correlation coefficient between the other features in $F_{ps}$ and the above feature is calculated. The features with correlation coefficients greater than 0.8 are removed and added to the subset in turn, instead of the above feature, and the classification accuracy is then calculated. The corresponding feature with the highest accuracy is retained in the feature subset and the corresponding classification accuracy is used to update the accuracy benchmark and complete the initialization. Subsequently, the most important feature in the current sequence is obtained from $F_{ps}$, and the features with a correlation greater than 0.8 are screened. The above features are added to the feature subset in turn, and the classification accuracy is calculated. The feature subset with the highest classification accuracy and greater than the current accuracy benchmark is retained. If the classification accuracy is below the current accuracy benchmark, no feature is added and no subsequent feature deletion step is performed. The least important feature is obtained from $F_{io}$ in turn, and the corresponding feature is deleted from the current feature subset. If the classification accuracy decreases, the above operation is withdrawn. Otherwise, the above operation is retained, and the accuracy benchmark is updated. The above process is iterated until the features in $F_{io}$ are traversed. The above process of adding and deleting features to and from the feature subset is repeated until $F_{ps}$ is empty. Finally, the optimal classification feature subset and the corresponding XGBoost detection model are obtained for preliminary detection.

#### 2.4.2. Correction of Preliminary Detection Results

Because the XGBoost detection model screens dynamic targets based on the differences between the multidimensional positions and geometric structures of the paired point cloud clusters in adjacent frame LiDAR point clouds, completely and effectively detecting dynamic targets with intermittent static states and first static and then moving states is impossible. Most LiDAR dynamic target online detection methods exhibit these problems. In addition, the optimal classification feature subset constructed in this paper underwent a feature screening step and dimensionality reduction step. Each feature is irreplaceable in the classification process. When the quantitative result of a feature is inaccurate, the XGBoost detection model may yield erroneous detection results. Aiming to address these two problems, a double Boyer–Moore voting-sliding window (DBMV−SW) based on the SW strategy [52] and the BMV strategy is designed to achieve the secondary correction of the preliminary XGBoost test results.

This paper holds that, in an actual situation, a change in motion state in all targets must be a gradual process. A target will not suddenly appear dynamic at a certain moment when its motion state is static, nor will a target suddenly appear static at a certain moment when its motion state is dynamic. In addition, the interframe position change of a point cloud cluster scanned by a dynamic target with an intermittent static state is also a gradual change process during the change in motion state. Therefore, aiming to identify and correct the anomaly detection from the preliminary detection results of XGBoost, the SW strategy is adopted, the point cloud of the i-th frame is taken as the research object, and the five consecutive historical point clouds of frame i−2,i−3,i−4,i−5,i−6 are taken as the sliding window range in this paper. The double registration method of the point cloud is used to process the geometric center point set of the point cloud clusters of the i-th frame and each frame point cloud in the window, and the motion state of the five nearest paired point cloud clusters of each point cloud cluster in the window of the i-th frame is obtained. The BMV strategy is used for the first vote for the above motion state corresponding to each point cloud cluster. If the preliminary detection result of adjacent frames is static but the voting result is dynamic, the preliminary detection result of adjacent frames is changed to dynamic. If both the initial detection result of the adjacent frame and the voting result are static, but the motion state of the nearest point cloud cluster with any two consecutive frames or more than two frames in the window is dynamic, the initial detection result of the adjacent frame is changed to dynamic. If the initial detection result of the adjacent frame is dynamic but the voting result is static, this frame must be judged in depth. The first two cases effectively detect dynamic targets with intermittent static states, and the third case effectively detects dynamic targets with static states and then moving states. The preliminary detection results of adjacent frames do not need to be modified in any case besides the above three cases.

Considering the third situation, which needs to be evaluated in depth, the X-axis displacement $D_{X}$, Y-axis displacement $D_{Y}$, and Z-axis displacement $D_{Z}$ are taken as dependent variables, and the arrangement number of each frame point cloud in the window is taken as an independent variable to fit the corresponding change trend. Because of the small amount of data and to ensure the efficiency of the data processing, the first-order linear function is used to fit the change trend of the above characteristics. Whether the current adjacent frames $D_{X}$, $D_{Y}$, and $D_{Z}$ conform to the corresponding change trend is verified as follows:

(13) $$\Delta C^{k}=f\left(\psi \right)-C^{k},\psi =6$$

In the formula, $\Delta C^{k}$ and $C^{k}$ represent the fitting error and quantization result of the k-th feature, respectively, and $f\left(\psi \right)$ represents the corresponding fitting function. Because the capacity of the window is 5, when judging whether the features of the current adjacent frame conform to the corresponding feature change trend, the independent variable ψ of the fitting function is 6. When $\Delta C^{k}>\sigma^{k}$, the detection result is not consistent with the change trend, and the ‘0’ fitting label is given; otherwise, the ‘1’ fitting label is given. σ represents the standard deviation of the corresponding feature in the window. In addition, the corresponding slope label is also given using the slope of the fitting function corresponding to each feature. If the absolute value of the slope is greater than 1, it is labelled ‘1’, and if the absolute value is less than 1 and greater than 0, it is labelled ‘0’.

The second vote is based on the fitting label and slope label corresponding to the three features. When a fitting label of ‘1’ corresponds to any feature, if the slope label ‘1’ corresponding to the above features is the majority label, the preliminary detection result of the adjacent frame does not need to be changed. In addition, any label combination must change the preliminary detection result of the adjacent frame to be static. The center point global coordinates of all the point cloud clusters that do not require changing the initial detection results of the adjacent frames during the detection process are recorded to form a set W. All the adjacent retrieval point cloud clusters belonging to W in the global coordinate system are regarded as dynamic point cloud clusters.

## 3. Experiments and Analysis

### 3.1. Source of Experimental Data

To verify whether the SCC−BSDR effectively considers the classification accuracy and model training efficiency of XGBoost, six datasets containing only quantitative features are randomly selected from the UCI machine learning database [53] (http://archive.ics.uci.edu/ accessed on 9 August 2023) as test data, including the wine, wireless indoor locating, iris, banknote authentication, abalone, and EEG eye state datasets.

To prevent overfitting by the training results of the LiDAR dynamic target detection model, the number of dynamic target samples and the number of static target samples contained in the training set should not differ by more than 200%. In this paper, an experimental platform is built based on LiDAR and Inertial Measurement Unit (IMU). LiDAR is used to collect the data for constructing the training set of the model in this paper, and IMU is used to collect the data needed for the comparison method. Using the above experimental platform, experimental data are collected from five real indoor and outdoor scenes, including an indoor warehouse scene with a lower people flow, an indoor teaching building scene with a higher people flow, an outdoor playground scene with a higher people flow, an outdoor campus trunk road scene with a higher traffic flow, and an indoor underground garage scene with a lower traffic flow. In addition, five common real indoor and outdoor scenes are selected as test scenes to verify the effectiveness of DBMV−SW and the detection effect of our method on LiDAR dynamic targets.

### 3.2. Experimental Platform and Experimental Scenes Overview

**Figure 5:**
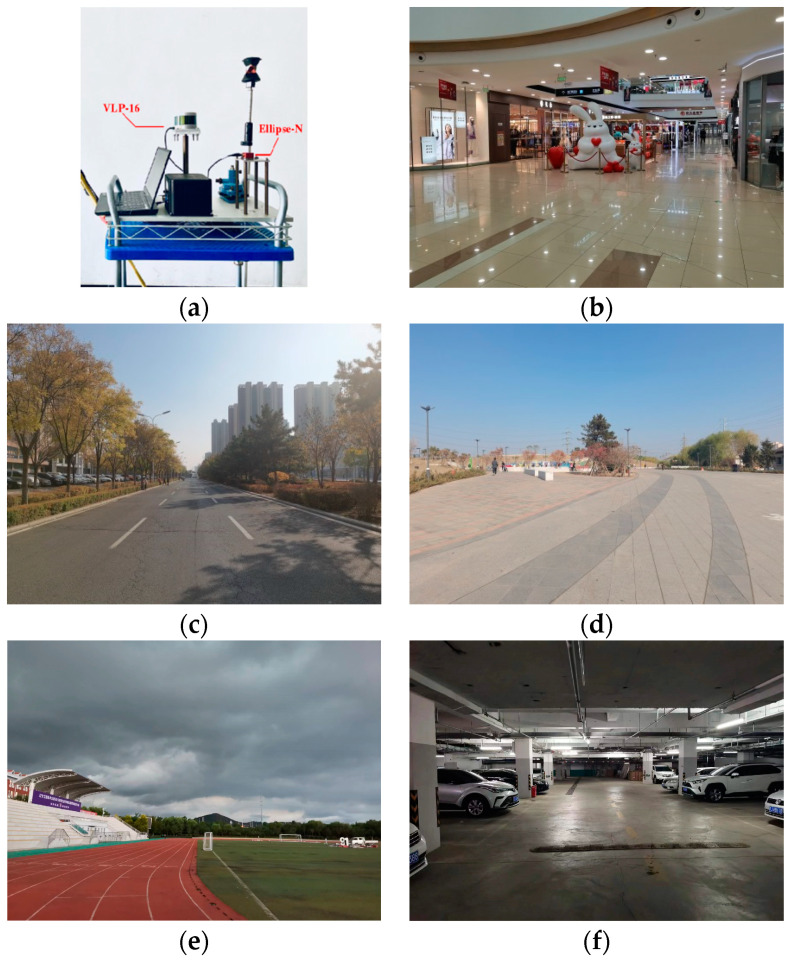
Experimental platform and scenes: (**a**) experimental platform; (**b**) experimental scene 1; (**c**) experimental scene 2; (**d**) experimental scene 3; (**e**) experimental scene 4; and (**f**) experimental scene 5.

**Figure 6:**
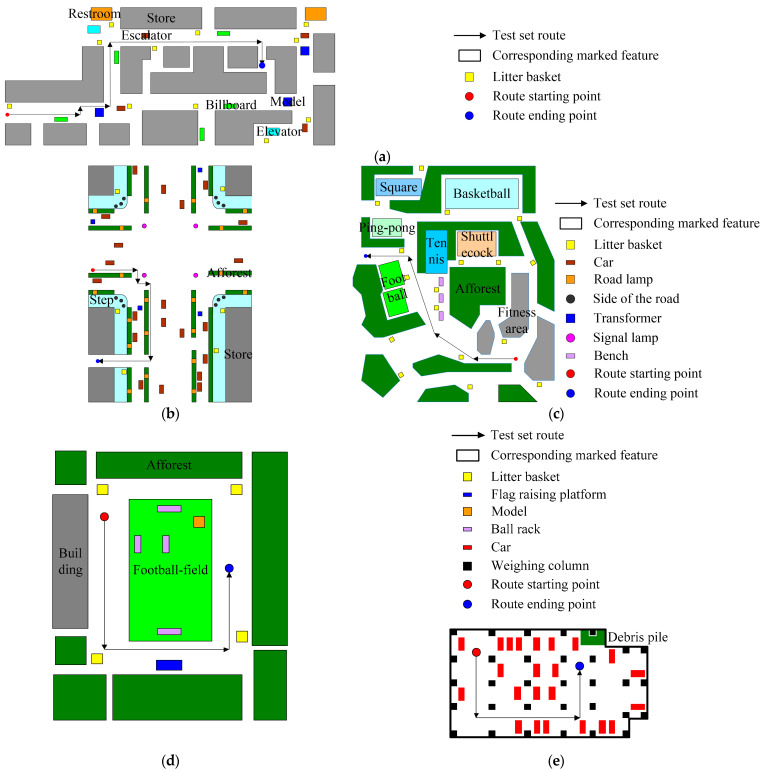
Experimental scene floor plans and platform motion trajectories: (**a**) experimental scene 1; (**b**) experimental scene 2; (**c**) experimental scene 3; (**d**) experimental scene 4; and (**e**) experimental scene 5.

The experimental platform is shown in Figure 5a. LiDAR uses Velodyne’s VLP−16, which has a horizontal scanning field of view of 360°, a vertical scanning field of view of 30°, and collects data at a frequency of 10 Hz. The IMU uses Ellipse−N from the SBG. The bias stability and repeatability of the gyroscope are 0.1 deg/s, the bias stability and repeatability of the accelerometer are 5 mg and 0.6 mg, respectively, and the data are collected at a frequency of 100 Hz. Test scene 1 is an indoor shopping mall, as shown in Figure 5b, which is distributed with static features such as models, stairs, and billboards. Test scene 2 is an outdoor bustling road section, as shown in Figure 5c. The road surface is flat, and there are shops on both sides. Between the shops and the motor vehicle lanes, there are steps, auxiliary roads, and green belts, and static features such as trash cans, isolation piers, street lamps, and substation boxes are distributed throughout the scene. Test scene 3 is an outdoor square, as shown in Figure 5d. This scene is distributed with static features such as trash cans, benches, and fitness equipment. Test scene 4 is an outdoor playground, as shown in Figure 5e. There are static features such as ball racks, models, a flag raising platform, and trash cans inside the site. Test scene 5 is an underground parking lot, as shown in Figure 5f. Static features such as load-bearing columns, fire hydrants, and debris piles are distributed inside the site. In scene 1, the dynamic targets are mainly pedestrians, and the people flow is high. Due to the indoor scene, the static objects are seriously occluded by dense dynamic targets to verify whether the target motion state detection effect of our method in the indoor environment is robust. The dynamic targets in scene 2 are mainly pedestrians and cars, which also have a high flow. Due to the outdoor environment and the existence of large dynamic targets, the occlusion problems of dynamic targets to static objects and between dynamic targets are more serious in this scene to verify whether the dynamic target detection effect of our method in complex outdoor environments is robust. The dynamic targets in scene 3 are mainly pedestrians. Although the flow of people is high, no large dynamic target exists, and this scene is outdoor and open. Therefore, the occlusion problem between targets can be ignored to verify whether the dynamic target detection effect of our method in an open outdoor environment is robust. The dynamic targets in scene 4 are mainly pedestrians. Similarly, the people flow is high and belongs to the outdoor open scene, but it belongs to the rainy weather. It is used to verify whether the target motion state detection effect of our method in a non-ideal outdoor environment is robust. In scene 5, the dynamic targets are mainly pedestrians and cars with less traffic, which belongs to the weak illumination scene. It is used to verify whether the target motion state detection effect of our method in a non-ideal indoor environment is robust. The plane structure of the above test experimental scene and the data acquisition route of the test set are shown in Figure 6a–e. The ground object categories represented by each symbol in the figure are shown in the legend.

### 3.3. Validation Experiment of the SCC−BSDR and Analysis

To verify whether the SCC−BSDR leads traditional XGBoost to account for both training efficiency and classification accuracy, for each dataset obtained from the UCI machine learning database, 70% of the data are used as the model training set, and the precision, recall, F1-measure, accuracy, and training time (Time) of the improved XGBoost algorithm on each test set are calculated. Based on the concept of ablation experiments, four algorithms are designed and compared with the algorithm in this paper (number 5):

- Algorithm 1: Forward search XGBoost algorithm based on weight
- Algorithm 2: Backward search XGBoost algorithm based on weight
- Algorithm 3: Bidirectional search XGBoost algorithm based on weight
- Algorithm 4: Bidirectional search XGBoost algorithm based on weight (including SCC−BS)
- Algorithm 5: Bidirectional search XGBoost algorithm based on weight (including SCC−BSDR) (our algorithm)

**Figure 7:**
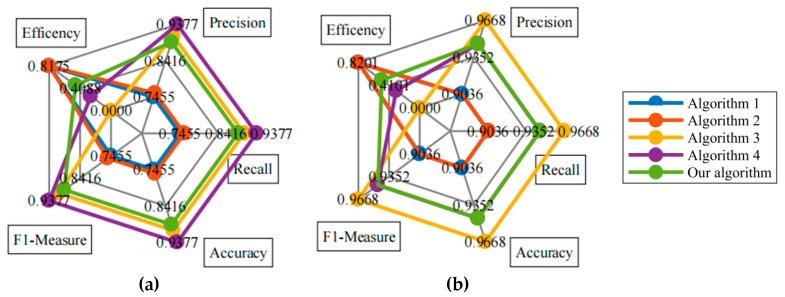
Comprehensive evaluation radar charts of each algorithm: (**a**) EEG eye state dataset; and (**b**) wine dataset.

Algorithm 3 is compared with algorithm 1–2 to verify the effectiveness of the BS strategy; our algorithm is compared with algorithm 3 to verify the effectiveness of the SCC−BSDR strategy; and our algorithm is compared with algorithm 4 to verify the effectiveness of the DR strategy. The F1-measure and Time corresponding to each algorithm are shown in Table 1. In addition, taking the EEG Eye State and Wine datasets as examples, the effectiveness of our algorithm is intuitively illustrated through radar charts, as shown in Figure 7. The training efficiency index Efficency in the figure is calculated as follows:

(14) $$Efficency=\left(Time_{max}-Time\right)/Time_{max}$$

where $Time_{max}$ represents the maximum value of the Time index of each algorithm corresponding to the current dataset.

Intuitively comparing the areas surrounded by the corresponding closed curves of each algorithm in Figure 7 shows that, on the two datasets, the area corresponding to our algorithm is always the largest, indicating that our algorithm can consider both model classification accuracy and training efficiency for quantitative datasets. The above viewpoint can be further confirmed by the indices shown in Table 1. The F1-measures of Algorithm 1 on the wine, iris, abalone, and banknote authentication datasets are greater than 0.9; the F1-measures of Algorithm 2 are greater than 0.9 only on the wine, abalone, and banknote authentication datasets; and the F1-measures of Algorithm 3, Algorithm 4, and our algorithm are greater than 0.89 and generally greater than 0.9 on each dataset. The training time required by the above three algorithms is greater than that required by algorithm 1 or algorithm 2, but the average training efficiency of our algorithm is 50.38% and 32.89% greater than that of algorithm 3 and algorithm 4, respectively. In addition, algorithm 3 and algorithm 4 achieve similar detection accuracies. The average classification accuracy of our algorithm is 1.88% and 2.05% lower than that of algorithm 3 and algorithm 4, respectively, but the efficiency improvement and accuracy reduction ratios are 26.7979 and 16.0439, respectively. Comprehensive analysis reveals that, although the model with the one-way search strategy achieves a high training efficiency, this model easily falls into the local optimal solution. The detection accuracies of algorithm 3, algorithm 4, and our algorithm are significantly higher than those of the other algorithms because of the bidirectional feature search strategy. Algorithm 4 uses the SCC−BS strategy to accelerate the construction of feature subsets, so it achieves a higher training efficiency than algorithm 3. Our algorithm uses the SCC−BSDR strategy to accelerate the construction of feature subsets and reduce their dimensionality. The optimal classification feature subset required for our algorithm to construct the classification decision tree is simpler than that for algorithm 4. Although the classification accuracy of a small part of the model is sacrificed, the model training efficiency is significantly improved. In summary, the effectiveness of SCC−BSDR is successfully verified by considering both classification accuracy and training efficiency.

### 3.4. Validation Experiment of the DBMV−SW and Analysis

In this paper, the DBMV−SW algorithm is proposed to correct the preliminary detection results of LiDAR dynamic targets based on the improved XGBoost algorithm. This compensates for the mechanicality of the machine learning algorithm and effectively detects dynamic targets with intermittent static states and first static and then moving states, improving the final detection accuracy. Since scene 2 contains many dynamic targets with the above special states, the data collected in this scene are used as test data and a DBMV−SW validity verification experiment is designed.

The LiDAR dynamic target detection method based on multidimensional features and the detection method excluding DBMV−SW are used to detect the dynamic target of each frame of the LiDAR point cloud in the dataset, and the static point cloud map is used as a reference. The number of static target benchmarks $NUM_{s}$, the number of dynamic target benchmarks $NUM_{d}$, the number of static target false detections $num_{s}$, and the number of dynamic target missed detections $num_{d}$ of all frame point clouds and each frame point cloud in the dataset are counted. The overall dynamic target correct detection rate $\eta_{d}$ and the static target error detection rate $\eta_{s}$ of all frame point clouds are calculated by Formula (15), as shown in Table 2. According to the total number of point cloud frames contained in the dataset and the total processing time, the time required to process a frame of point cloud is calculated, which is recorded as the average detection efficiency time, and the real-time measurement index $\eta_{t}$ is calculated by Formula (15), as shown in Table 2.

(15) $$\begin{array}{ccc} \eta_{s}=num_{s}/NUM_{s} & \eta_{d}=num_{d}/NUM_{d} & \eta_{t}=1-\left(time/0.1\right) \end{array}$$

In the formula, 0.1 represents the corresponding inter-frame time interval when the point cloud is collected at the frequency of 10 Hz.

**Figure 8:**
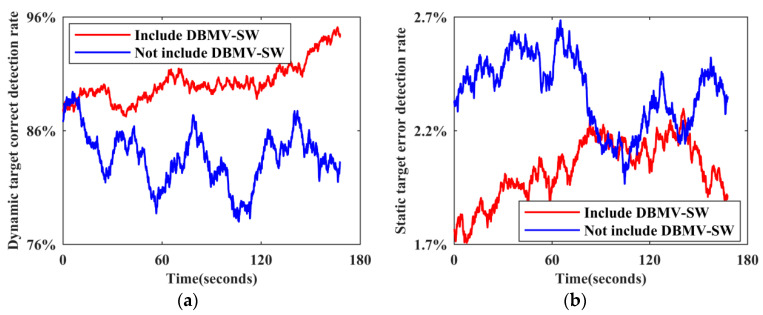
Time-varying sequence diagram of the detection effect: (**a**) dynamic target correct detection rate; and (**b**) static target error detection rate.

To visually compare the detection effects of the two methods, the dynamic target correct detection rate and the static target error detection rate corresponding to each frame point cloud are calculated by Formula (15), and time-varying sequence diagrams of the detection effects of the two methods are drawn, as shown in Figure 8.

As shown in Figure 8, after the secondary correction of the preliminary detection results by DBMV−SW, the dynamic target correct detection rate is generally above 0.85. Although the detection accuracy of individual frames is low, it is higher than the average detection accuracy without correction. In addition, the static target error detection rate is lower than 0.025, which is better than that in the case without correction. Combining this figure with Table 2 for a comprehensive analysis reveals that, after using the DBMV−SW strategy, the number of dynamic target missed detections is 40.29% lower and the number of static target false detections is 14.34% lower. Since DBMV−SW contains steps such as first voting, building tags, and second voting, and each step must be executed in a fixed order, it consumes part of the operation time on the basis of the preliminary detection based on the improved XGBoost, resulting in the average detection efficiency reduction of 22.48% and a real-time measurement index reduction of 7.80%. However, the ratio of the overall detection accuracy improvement to the efficiency reduction is 1.59, indicating that a small part of efficiency can be sacrificed to improve accuracy. The effectiveness of DBMV−SW is thus successfully verified.

### 3.5. Experiment of LiDAR Dynamic Target Detection Based on Multidimensional Features

Because the LiDAR point cloud in the dataset of this paper is dense, the visibility-based method is not suitable for dynamic target detection. In addition, voxel-based methods are all postprocessing methods. The process of constructing the reference benchmark for the training set employs this kind of method; as a result, it cannot be used as a comparison method. Therefore, two open-source segmentation-based methods, including a traditional clustering segmentation method and a learning segmentation method, are selected as comparison methods. Specifically, the LiDAR dynamic point cloud detection method based on calculating the similarity scores of the corresponding clusters in adjacent frames described in Reference [41] is selected as comparison method 1, and the deep-learning-based LiDAR dynamic point cloud detection method described in Reference [14] is selected as comparison method 2. Based on the data collected in three test scenes, the LiDAR dynamic target detection method based on multidimensional features proposed in this paper is comprehensively evaluated by comparing the detection accuracy and detection efficiency of these methods.

**Figure 9:**
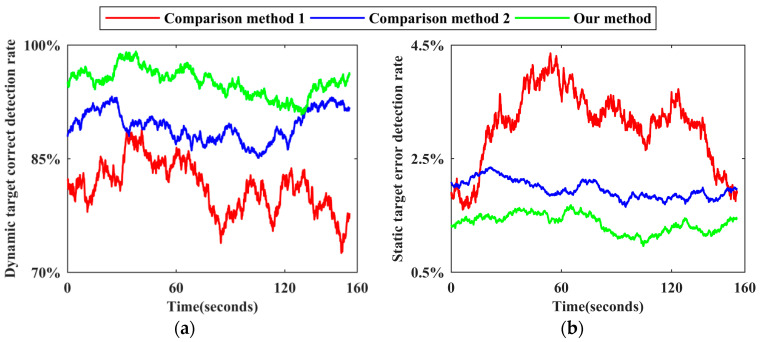
Time-varying sequence diagram of the detection effect of the three methods in scene 1: (**a**) dynamic target correct detection rate; and (**b**) static target error detection rate.

**Figure 10:**
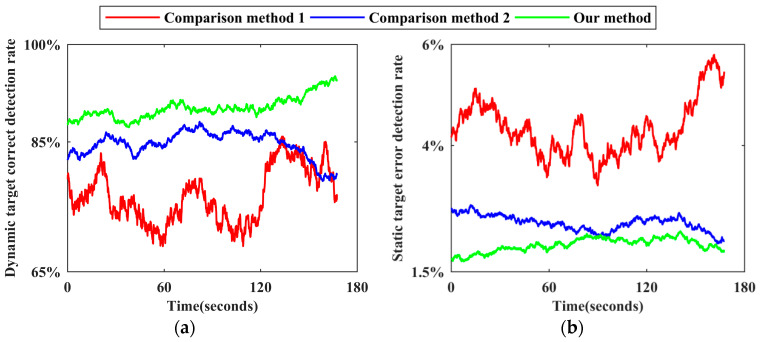
Time-varying sequence diagram of the detection effect of the three methods in scene 2: (**a**) dynamic target correct detection rate; and (**b**) static target error detection rate.

**Figure 11:**
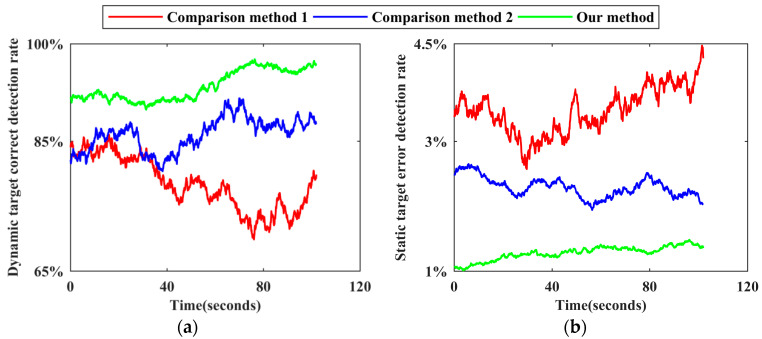
Time-varying sequence diagram of the detection effect of the three methods in scene 3: (**a**) dynamic target correct detection rate; and (**b**) static target error detection rate.

**Figure 12:**
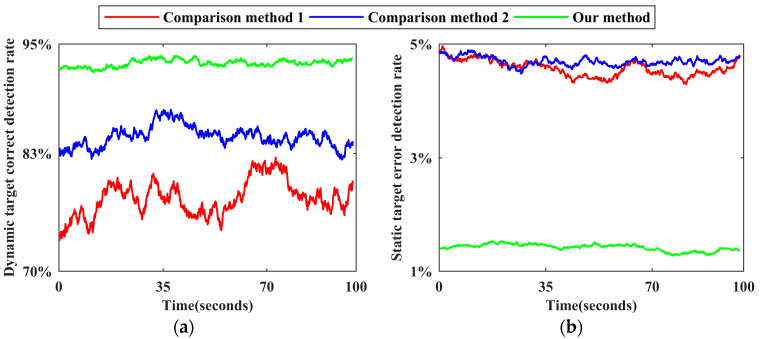
Time-varying sequence diagram of the detection effect of the three methods in scene 4: (**a**) dynamic target correct detection rate; and (**b**) static target error detection rate.

**Figure 13:**
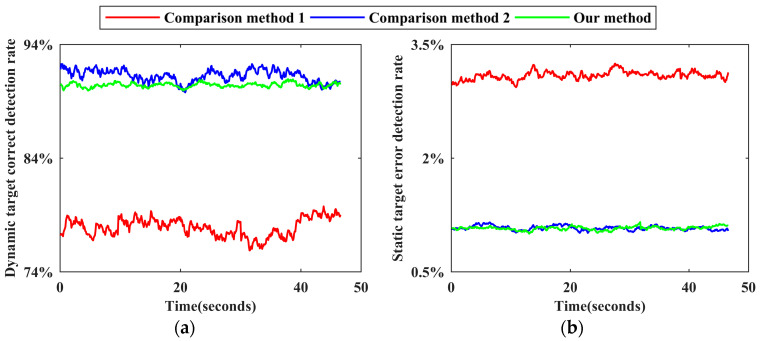
Time-varying sequence diagram of the detection effect of the three methods in scene 5: (**a**) dynamic target correct detection rate; and (**b**) static target error detection rate.

Using the reference benchmark of each scene, the number of static target benchmarks and the number of dynamic target benchmarks of each frame point cloud and all frame point clouds in the corresponding dataset are counted, respectively, as are the number of static target false detections and the number of dynamic target missed detections for each method. Following the calculation method of the dynamic target correct detection rate and the static target error detection rate of each frame point cloud described in Section 3.4, time−varying sequence diagrams of the corresponding detection effects of the three methods in each scene are drawn, as shown in Figure 9, Figure 10, Figure 11, Figure 12 and Figure 13. The overall dynamic target correct detection rate, static target error detection rate, detection efficiency, real-time measurement index, and mean values of the above indicators of each method on each dataset are calculated, as shown in Table 3. In addition, the dynamic target is regarded as the positive sample, and the static target is regarded as the negative sample. The overall target motion state detection Accuracy, Precision, Recall, F1-Measure, and mean values of the above indicators of each method on each dataset are calculated, as shown in Table 3.

Comparison method 1 judges the motion state of the target based on the change in the velocity and position of the adjacent frame paired point cloud cluster. For dynamic targets in intermittent static states and those first in static and then dynamic states, this method cannot effectively detect the above targets in static stages through only the adjacent frame detection mode. Therefore, as shown in Figure 9a, Figure 10a, Figure 11a, Figure 12a and Figure 13a the dynamic target correct detection rates in each scene are not high, and the detection effects in scene 2 and scene 5 are the worst, mainly because the above scenes contain a large number of special dynamic targets. The detection effect in scene 4 is slightly lower. Due to the rainy weather, there is an additional systematic error in the LiDAR point cloud measurements, and the inter-frame distribution of the error is irregular. Therefore, it directly affects the calculation accuracy of the velocity and position variation between the paired point cloud clusters and indirectly affects the detection effect. In addition, because comparison method 1 requires setting the heuristic threshold as the benchmark to judge the target motion state, the default threshold has limited applicability to different environments and scenes, resulting in a low detection robustness. Therefore, the trend of the detection results, as shown by the red line in all subgraphs over time, generally fluctuates greatly, and the lines corresponding to different scenes greatly differ.

Comparison method 2 trains the dynamic target detection network in an unsupervised mode. Although this approach can successfully detect some dynamic targets with the above special motion states, it is limited by the network structure and cannot effectively judge the motion state of the occluded target, and the detection effect decreases as the laser ranging length increases. Therefore, as shown in Figure 9a, Figure 10a, Figure 11a, Figure 12a and Figure 13a, the dynamic target correct detection rates in each scene are also not high, but the detection results are generally better than those of comparison method 1, because there is no need to set the heuristic threshold. Among them, due to the serious occlusion problem between the dynamic targets in scene 2, the corresponding detection effect of this scene is the worst. For scene 4, since the detection network trained by this method directly detects the dynamic target in the single frame point cloud, the above absolute detection mode is sensitive to the quality of point cloud data. Therefore, when environmental factors lead to a decrease in point cloud quality, there are more error detection phenomena. This method has the best detection effect in scene 5. Since there are fewer people in this scene, the occlusion between the targets is less, and the LiDAR measurement performance is not affected by the illumination conditions, the trained detection network can effectively identify most stationary cars and judge them as dynamic targets. As shown in Figure 9b, Figure 10b, Figure 11b, Figure 12b and Figure 13b, the static target detection effect of comparison method 2 is relatively stable, but except for scene 5, many point clouds are generated by dynamic target scanning in the retained static point cloud in other scenes, which does not effectively improve the quality of the LiDAR point cloud data.

As shown in Figure 9a, Figure 10a, Figure 11a, Figure 12a and Figure 13a, for five different indoor and outdoor scenes, the dynamic target detection accuracy of our method is greater than 85%, and the trend of the line is relatively stable, indicating that our method has a high detection accuracy and robustness for dynamic targets. Among them, the missed detection frequency of dynamic targets is higher in scene 2 and scene 5 than in the other scenes, primarily because our method cannot effectively detect dynamic targets that are always in static states during the data acquisition phase. Compared with the reference benchmark of the above scenes, static vehicles or temporary stalls existing in the data acquisition process are regarded as dynamic targets, but these dynamic targets are not detected by our method. Although the above situation also occurs in the other scenes, the number of these targets is limited in those scenes, so the detection accuracy is not significantly impacted. In addition, because our method comprehensively judges the motion state of point cloud clusters by calculating the multi-dimensional position and geometric structure differences of adjacent frame paired point cloud clusters, the above relative detection mode is less affected by environmental factors and the multi-dimensional features ensure the robustness of the detection process. Therefore, in scene 4, our method still obtains effective detection results. As shown in Figure 9b, Figure 10b, Figure 11b, Figure 12b and Figure 13b, since our method comprehensively considers the multi-dimensional information to detect the target motion state and performs a secondary correction, it can be effectively applied to a situation where dynamic targets block each other and dynamic targets block static targets in the environment. Therefore, the static target error detection rate of our method in each scene is relatively stable and generally below 2%. As shown in Figure 13b, the static target error detection rate of our method is generally below 1.1% in scene 5, and the overall trend is stable, indicating that our method can still maintain a robust detection ability under weak illumination conditions. Because the static target base in the actual environment is large, when the static target error detection rate is low, its impact on the subsequent corresponding point cloud processing work can be ignored.

The reasons for the ups and downs of the change trend of each broken line in Figure 9, Figure 10, Figure 11, Figure 12 and Figure 13 are analyzed as follows. Due to the large flow of people or vehicles in scene 1, scene 2, and scene 4, other targets will appear near the experimental platform at any time. The above targets will block part of the laser beam, resulting in the loss of more occluded other targets in the point clouds at this stage. The corresponding numbers of dynamic targets and static targets are greatly different from those at other times, which leads to the fact that the dynamic target correct detection rate and the static target error detection rate in Figure 9, Figure 10 and Figure 12 generally fluctuate greatly with time. Compared with the above three scenes, due to the small flow of people and vehicles in scene 5, the corresponding detection rate in Figure 13 is relatively stable with time, and the detection rate will change abruptly only in individual positions due to the change in the scanning field of view. As shown in Figure 6c, the environment in the first half of the data acquisition in scene 3 is relatively empty and secluded. When the experimental platform moves to the leisure area and the stadium in the second half, the corresponding detection rate in the second half of Figure 11 begins to fluctuate greatly with time due to the occlusion of other targets. In addition, due to the need to recover data acquisition equipment, the environment at the end of each data acquisition stage is relatively quiet and the dynamic target detection accuracy of the corresponding stage in each scene is generally relatively high.

After the above analysis of the limitations of the three methods and their detection performances in five experimental scenes, the detection effects of the three methods are quantitatively compared in Table 3. Compared with the two comparison methods, the average dynamic target correct detection rate of our method is 17.83% and 5.95% greater, the average static target error detection rate is 61.46% and 42.80% lower, the average detection efficiency is 27.60% and 10.75% higher, and the average real-time measurement index is 19.41% and 5.37% higher. The dynamic target correct detection rate is 92.41%, the static target error detection rate is 1.43%, and the detection efficiency is 0.0299 s. In addition, compared with the comparison methods, the average target motion state detection Accuracy, Precision, Recall, and F1-Measure of our method are also significantly improved. The Accuracy reaches 95.55%, indicating that the target motion state detection accuracy of our method is high. Precision and Recall reach 98.30% and 92.41%, respectively, indicating that our method has a strong dynamic target detection ability and also can effectively identify static targets. F1-Measure reaches 95.26%, indicating that the comprehensive detection ability of our method for dynamic targets and static targets is relatively balanced and has a strong robustness. In summary, our method has universal applicability to different experimental scenes. It can efficiently and accurately detect LiDAR dynamic targets and effectively improve the quality of LiDAR point clouds by screening dynamic point clouds.

In addition, considering that experimental scene 2 has a large range and contains rich targets, the point cloud collected in this scene is used as the test dataset to deeply test the influences of the volume factor, rate factor, and distribution position factor of the targets on the detection effect of our method. Based on the cloud cluster feature quantification methods in Section 2.3.1, the volume of the point cloud clusters, the distance between the center point of the point cloud clusters and the origin points, and the motion rate of the center point between the adjacent frame paired point cloud clusters are further calculated. Based on the above indicators, each point cloud cluster in the test dataset is divided into nine categories according to the rules shown in Table 4. The dynamic target in each category is regarded as a positive sample and the static target is regarded as negative sample. The target motion state detection Accuracy, Precision, Recall, and F1-Measure of different categories are calculated, as shown in Table 5.

As shown in the quantitative results of the detection effect in Table 5, the difference between the detection effects of the above nine categories is generally small and the detection effects of the three categories corresponding to different volume factors are similar. In the categories of rate factor, only the detection effect of the first category is slightly worse, and in the categories of distribution position factor, only the detection effect of the third category is worse. The reasons for the analysis are as follows. Our method extracts twelve features to construct the point cloud cluster classification feature system, which covers one-dimensional to three-dimensional, length to volume, and other features. When judging the motion state of the point cloud clusters, the above features play comprehensive roles. Therefore, even if there are abnormalities in the quantitative results of individual features, our method can still obtain robust detection results, so the corresponding detection effects of each category are generally less different. Except for the three-axis displacement feature, the remaining features in the feature system of our method are quantified by calculating the relative change degree. Therefore, the factors related to the geometric structure of the point cloud cluster will not affect the detection effect of our method. As a result, the detection effect between the categories corresponding to different volume factors is similar. Since our method cannot effectively detect the dynamic targets that always remain stationary during whole data acquisition, and these targets belong to the first category of the rate factor, the corresponding detection effect is slightly worse. The point cloud clusters contained in the third category of the distribution location factors are distributed in a range greater than 30 m from the origin. This category of target is usually generated by the residual laser beam after occlusion by many close-range targets. Because the distance between the laser scanning beam continues to expand with the scanning distance, the probability of an abnormal occurrence of the corresponding twelve features of these targets after quantification is generally large, resulting in poor detection results. However, due to the small number of these targets and their minimal role in point cloud inter-frame registration, point cloud model construction, and other related work, not too much energy needs to be spent to improve the detection effect of such targets. In summary, the detection effect of our method for LiDAR target motion state is less affected by factors such as volume and rate, and has a strong robustness.

## 4. Conclusions and Discussion

### 4.1. Conclusions

Aiming at the problem of current LiDAR dynamic target detection methods requiring heuristic thresholding, indirect computational assistance, supplementary sensor data, or postdetection, this paper proposes a LiDAR dynamic target detection method based on multidimensional features to detect the motion states of LiDAR point clouds with high efficiency and high precision. The method is analyzed and summarized using relevant experimental results.

- In this paper, 12 kinds of point cloud cluster features are extracted and quantified from the perspective of differences between the multidimensional positions and geometric structures of adjacent frame paired point cloud clusters. These features include uniaxial displacement, uniaxial span change degree, biaxial projection area change degree, point cloud number change degree, and other information, and are selected to ensure that the constructed point cloud cluster classification feature system is comprehensive. By constructing an a priori point cloud map and using the descriptor comparison method based on the voxel-based method, the motion state classification label is automatically assigned to the point cloud cluster in batches, avoiding human error. Based on the above point cloud cluster classification feature system and classification label quantization method, an experimental platform is built to collect data from real indoor and outdoor scenes to construct the model training dataset. To ensure the universal applicability of the LiDAR dynamic target detection model, the training set acquisition scene in this paper contains five representative indoor and outdoor environments.
- The training dataset of the proposed model contains many samples and the point cloud cluster classification feature system contains many classification features. Therefore, to account for the accuracy and efficiency of the LiDAR dynamic target detection model, the SCC−BSDR strategy is applied to the XGBoost training process. The test dataset is obtained from the UCI machine learning database and an ablation experiment is designed to verify the effectiveness of the above strategy. The results show that our algorithm is superior to the comparison algorithms when considering both classification accuracy and training efficiency. SCC−BSDR ensures that our method has a wider applicability and improves the average model training efficiency by 50.38%.
- Through model training, the optimal classification feature subset and corresponding XGBoost detection model are obtained. Considering the mechanical nature of machine learning and the influence of special dynamic targets on detection, a DBMV−SW strategy is proposed to correct the preliminary detection results of XGBoost twice. An ablation experiment is also designed to verify the effectiveness of this strategy. The results show that, after DBMV−SW correction, the number of dynamic target missed detections is reduced by 40.29%, the number of static target false detections is reduced by 14.34%, and the detection efficiency is reduced by 22.48%. The ratio of the accuracy improvement rate to the efficiency reduction rate is 1.59, indicating that this strategy sacrifices a small part of the operation efficiency but obtains a large improvement in accuracy.
- To comprehensively evaluate the detection effect of our method on LiDAR dynamic targets, two open-source LiDAR dynamic target detection methods based on the segmentation-based method are selected for comparison. The results show that the dynamic target correct detection rate of our method is 92.41%, the static target error detection rate is 1.43%, and the detection efficiency is 0.0299 s. Compared with those of the other two methods, the dynamic target correct detection rate of our method is 17.83% and 5.95% higher, the static target error detection rate is 61.46% and 42.80% lower, the detection efficiency is 27.60% and 10.75% higher, the real-time measurement index is 19.41% and 5.37% higher, and other comprehensive evaluation indexes are also significantly improved. Since our method comprehensively considers the multidimensional features between paired point cloud clusters to detect the motion states of point cloud clusters and constructs an optimal point cloud cluster classification feature subset and detection model, the detection accuracy and efficiency have significant advantages, and it has universal applicability for different scenes or conditions.
- Usually, LiDAR can meet most of the work requirements by collecting point clouds at the frequency of 10 Hz, and the corresponding point cloud inter-frame time interval is 0.1 s. At present, the average detection efficiency of our method for a frame of point cloud is 0.0302 s. Therefore, our method can be used as a point cloud preprocessing module for various practical works based on point cloud data. It is used to filter out dynamic point clouds to improve data quality, so as to ensure that the subsequent corresponding work results are more reliable.
- The point cloud double registration method proposed in this paper can register the center point sets of the adjacent frame point cloud clusters and obtain an effective pose relationship. It can be used alone to provide the initial value of the pose relationship for the SLAM registration algorithm based on the whole point cloud, thereby accelerating the convergence speed and improving accuracy. In addition, the SCC−BSDR proposed in this paper can also be transplanted to other multi-dimensional feature-based machine learning methods to screen optimal feature subsets, thereby improving data processing efficiency and ensuring excellent results.

### 4.2. Discussion

In this section, according to the experimental results and the corresponding conclusions, the advantages, shortcomings, and factors affecting the detection effect of our method and future research directions are discussed.

- Compared with other existing LiDAR dynamic target detection methods, our method does not require setting heuristic thresholds or using auxiliary processes such as plane projection and grid division. Furthermore, our method can directly detect the motion state of each point cloud cluster in the LiDAR point cloud. Since our method comprehensively detects the motion state of the target based on the multi-dimensional position and geometric structure difference between the adjacent frame paired point cloud clusters, and the preliminary detection results are corrected for the second time, the detection effect of our method is less affected by factors such as the volume, rate, and distribution position of the targets, and it has a universal applicability to different environments. Even under non-ideal conditions such as severe target occlusion, a complex scene structure, or rainy weather, our method can still maintain a strong robustness.
- In addition to the composition of the classification feature system, the secondary correction effect of the DBMV−SW strategy is a key factor that determines the final detection accuracy and efficiency. By analyzing the factors that can affect the above correction effect, three main factors are identified in this paper: the number of special dynamic targets, the frequency of motion state changes, the occlusion between targets, and the fitting function error. Specifically, the greater the number of dynamic targets with an intermittent static state or a first static and then moving state, or the greater the frequency of motion state changes, the more frequently the corresponding fitting labels and slope labels of these kinds of point cloud clusters will change. This will result in a greater probability of errors in the voting link in the secondary correction; this probability can be weakened by optimizing the voting strategy. The more serious the occlusion between the targets or the more common the occlusion phenomenon is, the more point cloud clusters that must be corrected or judged in depth after the voting process, increasing calculation costs. The error of the fitting function has little effect and can be weakened by optimizing the form of the fitting function; however, the first-order linear function can meet the current accuracy requirements and ensure a high data processing efficiency.
- Although our method achieves a high detection accuracy and efficiency, it is unable to effectively detect dynamic targets that remain stationary during whole data acquisition. To address the above problem, in the future research work, semantic information can be added to the point cloud cluster during the training process and dynamic targets can be eliminated by semantic label assistance, but the data processing workload of the scheme is significantly increased. In addition, a suitable scheme can be designed to correct the detection results of our method afterwards, but it can only meet post-application requirements. How to ensure its real-time performance needs to be further studied.
- With an increase in point cloud acquisition frequency, the detection efficiency of our method limits its application ability. When the point cloud is collected at the frequency of 20 Hz, the corresponding point cloud inter-frame time interval is 0.05 s. Although it is greater than the time required for the detection of our method, if it is used as a preprocessing module for other work, the overall work may find it difficult to meet the real-time performance. To address the above problem, more efficient machine learning methods or an improved fitting function form and voting process in DBMV−SW are considered in future research work, so as to improve the computational efficiency of our method.
- At present, our method can be applied to non-ideal conditions such as weak illumination or rainy weather. However, for extreme weather conditions such as haze days, heavy rain days, and heavy snow days, due to the high density of water or impurities in the atmosphere, the measurement performance of LiDAR is greatly affected, which, in turn, greatly affects the quality of the original point cloud, which eventually leads to a decrease in the detection accuracy of our method or even making it unusable. In future research work, for the case of less influence, it is considered to add an atmospheric correction module to compensate for the measurement error, thereby improving the quality of the point cloud. For the case of large influence, we consider using Radio Detection and Ranging (Radar) with a stronger penetration to collect data, and use our method to fuse and solve, so as to obtain effective dynamic target detection results.

## Figures and Tables

**Table 1:** Comparison of the classification accuracy and training efficiency of each algorithm.

Dataset	Contrast Ratio	Algorithm 1	Algorithm 2	Algorithm 3	Algorithm 4	Our Algorithm
Wine	F1-measure	0.9036	0.9036	0.9668	0.9467	0.9467
	Time (seconds)	2.6583	2.7273	14.7758	10.1208	7.2954
Wireless Indoor Locating	F1-measure	0.8785	0.8785	0.9667	0.9869	0.9376
	Time (seconds)	3.2112	2.9591	18.5169	13.5056	9.0544
Iris	F1-measure	0.9474	0.8998	0.9474	0.9237	0.9259
	Time (seconds)	2.2724	1.8457	7.8570	5.9028	2.6787
Abalone	F1-measure	0.9265	0.9176	0.9609	0.9627	0.9454
	Time (seconds)	3.2858	3.2993	15.7363	11.8754	7.8578
Banknote Authentication	F1-measure	0.9269	0.9269	0.9562	0.9562	0.9506
	Time (seconds)	3.6781	3.3008	15.8974	12.6205	8.0695
EEG Eye State	F1-measure	0.7455	0.7559	0.9063	0.9377	0.8908
	Time (seconds)	5.9584	5.9702	32.6547	23.9361	17.3668
Mean Value	F1-measure	0.8881	0.8804	0.9507	0.9523	0.9328
	Time (seconds)	3.5107	3.3504	17.5730	12.9935	8.7204

**Table 2:** The influence of DBMV−SW on detection accuracy and efficiency.

Index Types	Our Method (without DBMV−SW)	Our Method
The total number of dynamic target missed detections	20,852	12,450
The total number of static target false detections	4420	3786
The dynamic target correct detection rate	83.67%	90.25%
The static target error detection rate	2.37%	2.03%
Detection efficiency (seconds)	0.0258	0.0316
Real-time measurement index	74.21%	68.42%

**Table 3:** Comparison of the detection effect and efficiency of the comparison method and our method.

Index Types	Experimental Scene	Comparison Method 1	Comparison Method 2	Our Method
The total number of dynamic target missed detections	Scene 1	12,286	6972	3230
	Scene 2	29,904	19,600	12,450
	Scene 3	27,150	17,527	8205
	Scene 4	19,320	13,340	6271
	Scene 5	4427	1756	1922
	Mean value	18,617	11,839	6416
The total number of static target false detections	Scene 1	2114	1346	934
	Scene 2	8056	4587	3786
	Scene 3	4000	2640	1475
	Scene 4	4335	4459	1347
	Scene 5	504	176	175
	Mean value	3802	2642	1543
The dynamic target correct detection rate	Scene 1	81.25%	89.36%	95.07%
	Scene 2	76.58%	84.65%	90.25%
	Scene 3	78.36%	86.03%	93.46%
	Scene 4	78.03%	84.83%	92.87%
	Scene 5	77.91%	91.24%	90.41%
	Mean value	78.43%	87.22%	92.41%
The static target error detection rate	Scene 1	3.08%	1.96%	1.36%
	Scene 2	4.32%	2.46%	2.03%
	Scene 3	3.47%	2.29%	1.28%
	Scene 4	4.57%	4.70%	1.42%
	Scene 5	3.09%	1.08%	1.07%
	Mean value	3.71%	2.50%	1.43%
The target motion state detection accuracy	Scene 1	89.27%	93.80%	96.90%
	Scene 2	87.92%	92.30%	94.83%
	Scene 3	87.06%	91.62%	95.98%
	Scene 4	87.06%	90.26%	95.83%
	Scene 5	86.44%	94.69%	94.23%
	Mean value	87.55%	92.53%	95.55%
The target motion state detection precision	Scene 1	96.18%	97.75%	98.52%
	Scene 2	92.39%	95.93%	96.82%
	Scene 3	96.09%	97.61%	98.76%
	Scene 4	94.06%	94.36%	98.38%
	Scene 5	96.87%	99.05%	99.04%
	Mean value	95.12%	96.94%	98.30%
The target motion state detection recall	Scene 1	81.25%	89.36%	95.07%
	Scene 2	76.58%	84.65%	90.25%
	Scene 3	78.36%	86.03%	93.46%
	Scene 4	78.03%	84.83%	92.87%
	Scene 5	77.91%	91.24%	90.41%
	Mean value	78.43%	87.22%	92.41%
The target motion state detection F1-measure	Scene 1	88.09%	93.37%	96.77%
	Scene 2	83.75%	89.94%	93.42%
	Scene 3	86.32%	91.46%	96.04%
	Scene 4	85.30%	89.34%	95.54%
	Scene 5	86.36%	94.98%	94.53%
	Mean value	85.96%	91.82%	95.26%
Detection efficiency (seconds)	Scene 1	0.0386	0.0315	0.0289
	Scene 2	0.0462	0.0357	0.0316
	Scene 3	0.0415	0.0348	0.0302
	Scene 4	0.0427	0.0345	0.0307
	Scene 5	0.0375	0.0309	0.0282
	Mean value	0.0413	0.0335	0.0299
Real-time measurement index	Scene 1	61.39%	68.48%	71.09%
	Scene 2	53.77%	64.26%	68.39%
	Scene 3	58.48%	65.19%	69.83%
	Scene 4	57.29%	65.51%	69.32%
	Scene 5	62.52%	69.13%	71.79%
	Mean value	58.69%	66.51%	70.08%

**Table 4:** Point cloud clusters classification rules.

Classification Indications	Category 1	Category 2	Category 3
Volume factor (m^3^)	[0, 2]	(2, 15)	[15, +∞]
Rate factor (m/s)	[0, 5]	(5, 10)	[10, +∞]
Distribution position factor (m)	[0, 15]	(15, 30)	[30, +∞]

**Table 5:** The detection effect of different categories in scene 2.

Classification Indications		Accuracy	Precision	Recall	F1-Measure
Volume factor	Category 1	94.46%	97.10%	89.04%	92.90%
	Category 2	94.96%	97.33%	90.06%	93.55%
	Category 3	95.09%	97.01%	90.73%	93.76%
Rate factor	Category 1	93.90%	96.81%	87.88%	92.13%
	Category 2	95.25%	97.05%	91.09%	93.97%
	Category 3	95.21%	97.11%	90.93%	93.92%
Distribution position factor	Category 1	95.34%	97.04%	91.32%	94.09%
	Category 2	94.80%	96.85%	90.14%	93.37%
	Category 3	92.82%	94.87%	87.03%	90.78%

## Data Availability

Data are contained within the article.
